# 2-(4-Fluoro­benzyl­idene)-*N*-(4-meth­oxy­benzylidene)-1,3,4-thia­diazol-2-amine

**DOI:** 10.1107/S160053681002221X

**Published:** 2010-06-18

**Authors:** Qiu He, Kang An, Peng Wang, Peng Yu, Rong Wan

**Affiliations:** a Department of Applied Chemistry, College of Science, Nanjing University of Technology, No. 5 Xinmofan Road, Nanjing 210009, People’s Republic of China

## Abstract

The title compound, C_16_H_12_FN_3_OS, was synthesized by the reaction of 5-(4-meth­oxy­phen­yl)-1,3,4-thia­diazol-2-amine and 4-fluoro­benzaldehyde. An intra­molecular C—H⋯S hydrogen bond results in the formation of two five-membered rings. In the crystal structure, inter­molecular C—H⋯N hydrogen bonding links the mol­ecules, forming a two-dimensional network.

## Related literature

For the biological activity of 1,3,4-thiadiazole derivatives, see: Nakagawa *et al.* (1996 ▶); Wang *et al.* (1999 ▶).
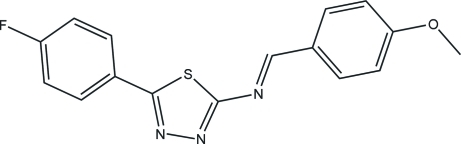


## Experimental

### 

#### Crystal data

C_16_H_12_FN_3_OS
*M* *_r_* = 313.35
Orthorhombic, 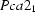

*a* = 7.4580 (15) Å
*b* = 17.821 (4) Å
*c* = 10.891 (2) Å
*V* = 1447.5 (5) Å^3^
*Z* = 4
Mo *K*α radiation
μ = 0.24 mm^−1^
*T* = 293 K
0.30 × 0.30 × 0.10 mm

#### Data collection

Enraf–Nonius CAD-4 diffractometer
Absorption correction: ψ scan (North *et al.*, 1968 ▶) *T*_min_ = 0.932, *T*_max_ = 0.977
2617 measured reflections
2617 independent reflections
1965 reflections with *I* > 2σ(*I*)
*R*_int_ = 0.045
3 standard reflections every 200 reflections  intensity decay: 1%

#### Refinement

*R*[*F* ^2^ > 2σ(*F* ^2^)] = 0.048
*wR*(*F* ^2^) = 0.132
*S* = 1.01
2617 reflections
199 parameters
1 restraint
H-atom parameters constrained
Δρ_max_ = 0.33 e Å^−3^
Δρ_min_ = −0.17 e Å^−3^
Absolute structure: Flack (1983 ▶), 1062 Friedel pairs
Flack parameter: −0.11 (13)

### 

Data collection: *CAD-4 EXPRESS* (Enraf–Nonius, 1989 ▶); cell refinement: *CAD-4 EXPRESS*; data reduction: *XCAD4* (Harms & Wocadlo, 1995 ▶); program(s) used to solve structure: *SHELXS97* (Sheldrick, 2008 ▶); program(s) used to refine structure: *SHELXL97* (Sheldrick, 2008 ▶); molecular graphics: *SHELXTL* (Sheldrick, 2008 ▶); software used to prepare material for publication: *SHELXL97*.

## Supplementary Material

Crystal structure: contains datablocks global, I. DOI: 10.1107/S160053681002221X/hg2691sup1.cif

Structure factors: contains datablocks I. DOI: 10.1107/S160053681002221X/hg2691Isup2.hkl

Additional supplementary materials:  crystallographic information; 3D view; checkCIF report

## Figures and Tables

**Table 1:** Hydrogen-bond geometry (Å, °)

*D*—H⋯*A*	*D*—H	H⋯*A*	*D*⋯*A*	*D*—H⋯*A*
C8—H8*A*⋯S	0.93	2.59	3.043 (5)	110
C12—H12*A*⋯S	0.93	2.75	3.138 (4)	106
C12—H12*A*⋯N3^i^	0.93	2.62	3.451 (6)	148

Symmetry code: (i) 
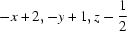
.

**Fig. 1.**
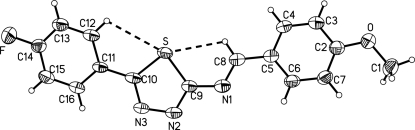
The molecular structure of the title molecule, with the atom-numbering scheme. Displacement ellipsoids are drawn at the 50% probability level. Hydrogen bonds are shown as dashed line.

**Fig. 2.**
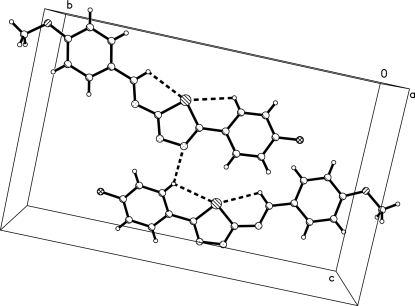
A partial packing diagram of(I). Hydrogen bonds are shown as dashed lines.

## supplementary crystallographic information

### Comment

1,3,4-Thiadiazole derivatives represent an interesting class of compounds possessing broad spectrum biological activities (Nakagawa *et al.*, 1996; Wang *et al.*, 1999). These compounds are known to exhibit diverse biological effects, such as insecticidal, fungicidal activities (Wang *et al.*, 1999).

We report herein the crystal structure of the title compound,(I). In the molecule of the title compound (Fig. 1), bond lengths are within normal ranges. Rings A(C2—C7), B(S/C9/N2/N3/C10) and C(C11—C16) are planar. The dihedral angle between them is A/B = 21.4 (1) Å, A/C=29.6 (3)Å and B/C= 10.7 (4) Å. The intramolecular C—H···S hydrogen bonds (Table 1) result in the formation of two planar five-membered rings D(H8A/C8/N1/C9/S) and E(S/H12A/C12/C11/C10). They are oriented with respect to the adjacent rings at dihedral angles of A/D= 11.6 (4) Å, B/D= 14.1 (4) Å, C/D= 24.5 (1) Å, A/E= 27.4 (1) Å, B/E= 6.1 (1) Å, C/E= 8.2 (1)Å and D/E= 19.5 (1) Å. In the crystal structure, intermolecular C—H···S hydrogen bond (Table 1) links the molecules to form a two-dimensional network (Fig. 2), in which they may be effective in the stabilization of the structure.

### Experimental

5-(4-methoxyphenyl)-1,3,4-thiadiazol-2-amine(5 mmol) and 4-fluorobenzaldehyde(5 mmol) were added in toluene (50 ml). The water was removed by distillation for 5 h. The reaction mixture was left to cool to room temperature, filtered, and the filter cake was crystallized from acetone to give pure compound (I) (m.p. 412–414 K). Crystals of (I) suitable for X-ray diffraction were obtained by slow evaporation of an acetone solution.

### Refinement

All H atoms bonded to the C atoms were placed geometrically at the distances of 0.93–0.97 Å and included in the refinement in riding motion approximation with *U*_iso_(H) = 1.2_eq_ of the carrier atom.

### Crystal data

**Table:**

C_16_H_12_FN_3_OS	*D*_x_ = 1.438 Mg m^−^^3^
*M**_r_* = 313.35	Melting point = 412–414 K
Orthorhombic, *P**c**a*2_1_	Mo *K*α radiation, λ = 0.71073 Å
Hall symbol: P 2ac -2ac	Cell parameters from 25 reflections
*a* = 7.4580 (15) Å	θ = 9–13°
*b* = 17.821 (4) Å	µ = 0.24 mm^−^^1^
*c* = 10.891 (2) Å	*T* = 293 K
*V* = 1447.5 (5) Å^3^	Block, colourless
*Z* = 4	0.30 × 0.30 × 0.10 mm
*F*(000) = 648	

### Data collection

**Table:**

Enraf–Nonius CAD-4 diffractometer	1965 reflections with *I* > 2σ(*I*)
Radiation source: fine-focus sealed tube	*R*_int_ = 0.045
graphite	θ_max_ = 25.3°, θ_min_ = 1.1°
ω/2θ scans	*h* = −8→0
Absorption correction: ψ scan (North *et al.*, 1968)	*k* = 0→21
*T*_min_ = 0.932, *T*_max_ = 0.977	*l* = −13→13
2617 measured reflections	3 standard reflections every 200 reflections
2617 independent reflections	intensity decay: 1%

### Refinement

**Table:**

Refinement on *F*^2^	Secondary atom site location: difference Fourier map
Least-squares matrix: full	Hydrogen site location: inferred from neighbouring sites
*R*[*F*^2^ > 2σ(*F*^2^)] = 0.048	H-atom parameters constrained
*wR*(*F*^2^) = 0.132	*w* = 1/[σ^2^(*F*_o_^2^) + (0.078*P*)^2^] where *P* = (*F*_o_^2^ + 2*F*_c_^2^)/3
*S* = 1.01	(Δ/σ)_max_ < 0.001
2617 reflections	Δρ_max_ = 0.33 e Å^−^^3^
199 parameters	Δρ_min_ = −0.17 e Å^−^^3^
1 restraint	Absolute structure: Flack (1983), 1062 Friedel pairs
Primary atom site location: structure-invariant direct methods	Flack parameter: −0.11 (13)

### Special details

**Table:**

Geometry. All e.s.d.'s (except the e.s.d. in the dihedral angle between two l.s. planes) are estimated using the full covariance matrix. The cell e.s.d.'s are taken into account individually in the estimation of e.s.d.'s in distances, angles and torsion angles; correlations between e.s.d.'s in cell parameters are only used when they are defined by crystal symmetry. An approximate (isotropic) treatment of cell e.s.d.'s is used for estimating e.s.d.'s involving l.s. planes.
Refinement. Refinement of *F*^2^ against ALL reflections. The weighted *R*-factor *wR* and goodness of fit *S* are based on *F*^2^, conventional *R*-factors *R* are based on *F*, with *F* set to zero for negative *F*^2^. The threshold expression of *F*^2^ > σ(*F*^2^) is used only for calculating *R*-factors(gt) *etc*. and is not relevant to the choice of reflections for refinement. *R*-factors based on *F*^2^ are statistically about twice as large as those based on *F*, and *R*- factors based on ALL data will be even larger.

### Fractional atomic coordinates and isotropic or equivalent isotropic displacement parameters (Å^2^)

**Table:**

	*x*	*y*	*z*	*U*_iso_*/*U*_eq_	
S	0.92308 (13)	0.41730 (5)	0.79211 (10)	0.0604 (3)	
O	0.9032 (5)	−0.04222 (18)	0.5620 (3)	0.0858 (10)	
F	0.8635 (5)	0.78682 (14)	0.8637 (3)	0.0990 (9)	
N1	0.8911 (5)	0.2684 (2)	0.8632 (3)	0.0645 (10)	
N2	0.8471 (5)	0.3651 (2)	1.0054 (3)	0.0687 (9)	
N3	0.8461 (5)	0.4421 (2)	1.0171 (3)	0.0654 (9)	
C1	0.9071 (7)	−0.1065 (3)	0.6422 (6)	0.0918 (16)	
H1B	0.9074	−0.1516	0.5940	0.138*	
H1C	0.8031	−0.1060	0.6942	0.138*	
H1D	1.0132	−0.1048	0.6920	0.138*	
C2	0.9047 (6)	0.0268 (3)	0.6164 (4)	0.0678 (12)	
C3	0.8810 (6)	0.0860 (2)	0.5372 (4)	0.0739 (12)	
H3B	0.8684	0.0770	0.4535	0.089*	
C4	0.8755 (6)	0.1585 (3)	0.5806 (4)	0.0687 (11)	
H4A	0.8579	0.1981	0.5262	0.082*	
C5	0.8963 (5)	0.1733 (2)	0.7064 (4)	0.0603 (10)	
C6	0.9228 (5)	0.1129 (2)	0.7838 (5)	0.0657 (10)	
H6A	0.9388	0.1217	0.8673	0.079*	
C7	0.9261 (6)	0.0401 (3)	0.7411 (4)	0.0715 (13)	
H7A	0.9425	0.0003	0.7952	0.086*	
C8	0.8852 (6)	0.2493 (3)	0.7494 (4)	0.0643 (11)	
H8A	0.8730	0.2871	0.6912	0.077*	
C9	0.8836 (5)	0.3439 (2)	0.8931 (4)	0.0574 (10)	
C10	0.8857 (5)	0.4763 (2)	0.9128 (3)	0.0542 (9)	
C11	0.8879 (5)	0.5579 (2)	0.9023 (3)	0.0512 (9)	
C12	0.9457 (5)	0.5931 (2)	0.7942 (5)	0.0611 (9)	
H12A	0.9893	0.5640	0.7298	0.073*	
C13	0.9392 (5)	0.6695 (2)	0.7818 (5)	0.0661 (10)	
H13A	0.9787	0.6924	0.7099	0.079*	
C14	0.8735 (6)	0.7118 (2)	0.8768 (4)	0.0673 (11)	
C15	0.8172 (6)	0.6802 (3)	0.9853 (4)	0.0709 (12)	
H15A	0.7758	0.7101	1.0492	0.085*	
C16	0.8234 (5)	0.6033 (2)	0.9975 (3)	0.0606 (10)	
H16A	0.7841	0.5813	1.0701	0.073*	

### Atomic displacement parameters (Å^2^)

**Table:**

	*U*^11^	*U*^22^	*U*^33^	*U*^12^	*U*^13^	*U*^23^
S	0.0706 (6)	0.0737 (6)	0.0370 (4)	−0.0041 (5)	0.0021 (5)	0.0022 (6)
O	0.120 (3)	0.064 (2)	0.073 (2)	0.0014 (18)	0.0043 (19)	0.0027 (16)
F	0.140 (2)	0.0664 (16)	0.091 (2)	−0.0069 (16)	−0.0093 (18)	0.0074 (14)
N1	0.064 (2)	0.077 (3)	0.052 (2)	−0.0011 (18)	−0.0054 (17)	0.0085 (18)
N2	0.082 (2)	0.084 (2)	0.0407 (18)	−0.0013 (19)	−0.0027 (17)	0.0109 (16)
N3	0.086 (2)	0.076 (2)	0.0349 (17)	−0.0007 (19)	0.0027 (16)	0.0037 (15)
C1	0.106 (4)	0.065 (3)	0.104 (4)	−0.001 (3)	0.014 (3)	0.013 (3)
C2	0.071 (3)	0.074 (3)	0.059 (3)	0.002 (2)	0.002 (2)	0.004 (2)
C3	0.094 (3)	0.079 (3)	0.049 (2)	0.005 (2)	−0.002 (2)	0.005 (2)
C4	0.085 (3)	0.070 (3)	0.051 (2)	0.002 (2)	0.000 (2)	0.0147 (19)
C5	0.061 (2)	0.070 (3)	0.050 (2)	−0.003 (2)	0.0007 (18)	0.0064 (19)
C6	0.075 (2)	0.074 (2)	0.048 (2)	0.0045 (19)	0.003 (2)	0.009 (3)
C7	0.080 (3)	0.071 (3)	0.063 (3)	0.012 (2)	0.000 (2)	0.014 (2)
C8	0.067 (2)	0.077 (3)	0.050 (2)	0.000 (2)	0.0010 (18)	0.0091 (19)
C9	0.057 (2)	0.073 (3)	0.0419 (19)	−0.0012 (19)	−0.0034 (16)	0.0054 (18)
C10	0.0435 (18)	0.085 (3)	0.0337 (18)	0.0011 (18)	−0.0032 (14)	0.0043 (17)
C11	0.0456 (19)	0.075 (3)	0.0328 (17)	−0.0029 (17)	−0.0051 (13)	0.0045 (18)
C12	0.059 (2)	0.086 (3)	0.0384 (16)	−0.0027 (18)	−0.003 (2)	0.003 (3)
C13	0.065 (2)	0.085 (3)	0.049 (2)	−0.010 (2)	−0.0044 (19)	0.006 (2)
C14	0.075 (3)	0.070 (3)	0.057 (3)	−0.004 (2)	−0.009 (2)	0.007 (2)
C15	0.081 (3)	0.082 (3)	0.049 (2)	0.005 (2)	−0.003 (2)	−0.007 (2)
C16	0.060 (2)	0.083 (3)	0.0388 (18)	0.001 (2)	0.0008 (18)	−0.0020 (18)

### Geometric parameters (Å, °)

**Table:**

S—C10	1.706 (4)	C4—H4A	0.9300
S—C9	1.734 (4)	C5—C6	1.381 (5)
O—C2	1.365 (6)	C5—C8	1.436 (6)
O—C1	1.441 (6)	C6—C7	1.379 (6)
F—C14	1.347 (5)	C6—H6A	0.9300
N1—C8	1.287 (6)	C7—H7A	0.9300
N1—C9	1.385 (5)	C8—H8A	0.9300
N2—C9	1.309 (5)	C10—C11	1.459 (6)
N2—N3	1.379 (5)	C11—C16	1.401 (5)
N3—C10	1.323 (5)	C11—C12	1.402 (7)
C1—H1B	0.9600	C12—C13	1.370 (5)
C1—H1C	0.9600	C12—H12A	0.9300
C1—H1D	0.9600	C13—C14	1.370 (7)
C2—C3	1.374 (6)	C13—H13A	0.9300
C2—C7	1.388 (6)	C14—C15	1.375 (6)
C3—C4	1.377 (6)	C15—C16	1.376 (6)
C3—H3B	0.9300	C15—H15A	0.9300
C4—C5	1.403 (6)	C16—H16A	0.9300
			
C10—S—C9	87.1 (2)	C2—C7—H7A	120.4
C2—O—C1	117.0 (4)	N1—C8—C5	124.2 (4)
C8—N1—C9	118.8 (4)	N1—C8—H8A	117.9
C9—N2—N3	112.0 (3)	C5—C8—H8A	117.9
C10—N3—N2	112.2 (3)	N2—C9—N1	120.5 (4)
O—C1—H1B	109.5	N2—C9—S	114.3 (3)
O—C1—H1C	109.5	N1—C9—S	125.2 (3)
H1B—C1—H1C	109.5	N3—C10—C11	122.0 (4)
O—C1—H1D	109.5	N3—C10—S	114.4 (3)
H1B—C1—H1D	109.5	C11—C10—S	123.5 (3)
H1C—C1—H1D	109.5	C16—C11—C12	118.0 (4)
O—C2—C3	114.8 (4)	C16—C11—C10	121.0 (3)
O—C2—C7	125.4 (4)	C12—C11—C10	121.0 (4)
C3—C2—C7	119.9 (4)	C13—C12—C11	121.1 (5)
C2—C3—C4	120.6 (4)	C13—C12—H12A	119.5
C2—C3—H3B	119.7	C11—C12—H12A	119.5
C4—C3—H3B	119.7	C14—C13—C12	119.0 (5)
C3—C4—C5	120.5 (4)	C14—C13—H13A	120.5
C3—C4—H4A	119.7	C12—C13—H13A	120.5
C5—C4—H4A	119.7	F—C14—C13	119.0 (4)
C6—C5—C4	117.8 (4)	F—C14—C15	118.8 (4)
C6—C5—C8	123.0 (4)	C13—C14—C15	122.2 (4)
C4—C5—C8	119.2 (4)	C14—C15—C16	118.7 (4)
C7—C6—C5	122.0 (4)	C14—C15—H15A	120.6
C7—C6—H6A	119.0	C16—C15—H15A	120.6
C5—C6—H6A	119.0	C15—C16—C11	121.0 (4)
C6—C7—C2	119.3 (4)	C15—C16—H16A	119.5
C6—C7—H7A	120.4	C11—C16—H16A	119.5
			
C9—N2—N3—C10	−1.3 (5)	C10—S—C9—N2	−0.2 (3)
C1—O—C2—C3	−173.1 (4)	C10—S—C9—N1	−179.1 (4)
C1—O—C2—C7	6.2 (7)	N2—N3—C10—C11	178.3 (3)
O—C2—C3—C4	178.5 (4)	N2—N3—C10—S	1.1 (4)
C7—C2—C3—C4	−0.9 (7)	C9—S—C10—N3	−0.5 (3)
C2—C3—C4—C5	0.8 (7)	C9—S—C10—C11	−177.7 (3)
C3—C4—C5—C6	0.2 (6)	N3—C10—C11—C16	−9.2 (6)
C3—C4—C5—C8	−178.3 (4)	S—C10—C11—C16	167.7 (3)
C4—C5—C6—C7	−1.1 (6)	N3—C10—C11—C12	174.2 (4)
C8—C5—C6—C7	177.5 (4)	S—C10—C11—C12	−8.8 (5)
C5—C6—C7—C2	0.9 (7)	C16—C11—C12—C13	−0.1 (6)
O—C2—C7—C6	−179.2 (4)	C10—C11—C12—C13	176.6 (3)
C3—C2—C7—C6	0.1 (7)	C11—C12—C13—C14	−0.5 (6)
C9—N1—C8—C5	178.4 (3)	C12—C13—C14—F	−178.6 (3)
C6—C5—C8—N1	−3.1 (7)	C12—C13—C14—C15	1.4 (7)
C4—C5—C8—N1	175.4 (4)	F—C14—C15—C16	178.5 (4)
N3—N2—C9—N1	179.8 (3)	C13—C14—C15—C16	−1.5 (7)
N3—N2—C9—S	0.9 (5)	C14—C15—C16—C11	0.9 (6)
C8—N1—C9—N2	164.0 (4)	C12—C11—C16—C15	−0.1 (6)
C8—N1—C9—S	−17.2 (6)	C10—C11—C16—C15	−176.7 (4)

### Hydrogen-bond geometry (Å, °)

**Table:**

*D*—H···*A*	*D*—H	H···*A*	*D*···*A*	*D*—H···*A*
C8—H8A···S	0.93	2.59	3.043 (5)	110
C12—H12A···S	0.93	2.75	3.138 (4)	106
C12—H12A···N3^i^	0.93	2.62	3.451 (6)	148

Symmetry codes: (i) −*x*+2, −*y*+1, *z*−1/2.
